# Napping improves interhemispheric functional coordination during night shifts in nurses: analysis of voxel-mirrored homotopic connectivity

**DOI:** 10.3389/fneur.2026.1889180

**Published:** 2026-08-20

**Authors:** Li-Min Cai, Zhe-Yi Huang, Hong-Yu Lin, Xiao-Hong Zhang, Qiu-Yi Dong, Hua-Jun Chen, Yan-Juan Lin

**Affiliations:** 1 Department of Stomatology, Fujian Medical University Union Hospital, Fuzhou, China; 2 Department of Radiology, Fujian Medical University Union Hospital, Fuzhou, China; 3 Department of Nursing, Fujian Medical University Union Hospital, Fuzhou, China; 4 Department of Cardiovascular Surgery, Fujian Medical University Union Hospital, Fuzhou, China

**Keywords:** napping, night shift, nurse, resting-state functional magnetic resonance imaging, voxel-mirrored homotopic connectivity

## Abstract

**Purpose:**

Napping has been shown to improve cognitive performance in nurses working night shifts; however, the underlying mechanisms remain poorly understood. This study aims to investigate the effects of night shift napping on interhemispheric functional coordination and examine their relationship with cognitive changes in nurses.

**Methods:**

A total of 21 female nurses participated in three sessions: Rested Wakefulness (RW), Sleep Deprivation (SD), and Napping (NAP). During the RW session, participants were allowed normal sleep; during SD, they remained awake for 24 h; and in the NAP session, a two-hour nap was allowed during their night shift. Neurocognitive assessments and resting-state functional magnetic resonance imaging were conducted after SD and NAP sessions. Voxel-mirrored homotopic connectivity (VMHC) analysis was used to assess interhemispheric functional coordination.

**Results:**

Compared with RW, SD led to a significant increase in VMHC, primarily involving the bilateral temporal, occipital, and parietal cortices, sensorimotor areas, middle and inferior frontal cortices, insula, subcortical regions, and cerebellum [family-wise error (FWE)-corrected *p* < 0.05]. In contrast to SD, NAP resulted in a reduction of VMHC in a broad range of brain regions that exhibited increased VMHC during SD (FWE-corrected *p* < 0.05). The alterations in global VMHC after NAP were correlated with improved neurocognitive performance among the nurses (*p* < 0.05).

**Conclusion:**

Night shift napping appears to reorganize interhemispheric functional connections, which tentatively underpins its potential role in cognitive recovery from SD among nurses, with such neural-cognitive links necessitating further validation in expanded sample cohorts.

## Introduction

Shift work is essential for hospital nurses to ensure continuous patient care. However, prolonged night shifts can disrupt the circadian rhythm and exacerbate sleep deprivation (SD) (1). Research has demonstrated that SD from night shifts is associated with negative health outcomes, including metabolic syndrome and cardiovascular disease (2). Night-shift nurses perform worse on visual attention tests than day-shift nurses (3). Additionally, fatigue and cognitive impairment resulting from SD may lead to suboptimal clinical practice and compromise patient safety (4). Therefore, interventions are crucial to mitigate the adverse effects of SD associated with night shifts.

Napping (NAP) during a night shift is considered beneficial for workers, as it provides an opportunity to rest during periods of diminished alertness and performance (5). Previous research has shown that NAP can reduce fatigue and sleepiness (6) and lower the risk of medical errors (7) during night shifts. Additionally, night shift napping has been shown to improve cognitive performance (3, 8). However, the specific mechanisms by which napping restores cognitive function impaired by SD remain insufficiently understood.

Resting-state functional magnetic resonance imaging (rs-fMRI) has become a widely used method for investigating intrinsic brain function due to its noninvasive nature and ease of acquisition (9). Recent rs-fMRI studies have identified functional alterations in the brains of individuals with SD, including impaired functional connectivity between the thalamus and cortical regions, aberrant connectivity within the default mode network and the frontal–parietal network (10). Another study demonstrated that altered dynamic amplitude of low-frequency fluctuations, a measure reflecting the variability of local spontaneous neural activity, was observed in nurses experiencing night shift-related SD, which was correlated with changes in memory function performance (11). Furthermore, abnormalities in functional stability, a metric characterizing the stability of the brain’s functional architecture, have been identified in multiple regions associated with neurocognitive functions. These regions include the default mode network, the posterior and anterior lobes of the cerebellum, and the parahippocampal gyrus, particularly in women with SD (12). Despite evidence of SD-related brain alterations, it remains unclear whether napping can ameliorate these dysfunctions.

Interhemispheric coordination is essential for maintaining brain function (13). For example, homotopic functional connectivity is a key component of the brain’s intrinsic functional architecture and is associated with various cognitive functions, such as executive function and memory (14). Voxel-mirrored homotopic connectivity (VMHC), measured using rs-fMRI, quantifies interhemispheric coordination by calculating functional connectivity between a voxel and its contralateral voxel (15). VMHC has been shown to be sensitive for detecting abnormal interhemispheric functional coordination in neuropsychological conditions, including Alzheimer’s disease, autism, and schizophrenia (16, 17). Notably, a previous study found significantly increased VMHC in the thalamus after SD, which might have contributed to compensatory maintenance of cognitive performance (18). However, no studies have yet used VMHC to investigate whether interhemispheric functional connectivity is affected by NAP in individuals with SD.

An fMRI study has shown that a 90-min mid-afternoon nap can restore hippocampal function, which is positively correlated with enhanced memory consolidation (19). Based on this, we hypothesize that NAP during night shifts may help mitigate SD-related brain dysfunction. To test this hypothesis, we aim to: (1) examine the effects of SD and napping on interhemispheric coordination in nurses working night shifts using the VMHC approach; and (2) evaluate the correlations between changes in interhemispheric functional coordination and cognitive performance.

## Materials and methods

### Subjects

This study was approved by the Research Ethics Committee of Fujian Medical University Union Hospital. Informed consent was acquired from all participants prior to the commencement of the study. A total of 21 young female nurses (mean age: 20.1 ± 0.6 years; mean years of education: 14.4 ± 1.5 years) participated in the study. All participants were right-handed. Nurses were excluded if they met any of the following criteria: (1) a history of sleep disorders or other neuropsychiatric conditions, (2) current use of psychotropic medications, (3) uncontrolled endocrine or metabolic disorders (e.g., thyroid dysfunction), serious medical conditions (e.g., heart failure or cancer), or (4) contraindications for undergoing MRI.

**Figure 1:**
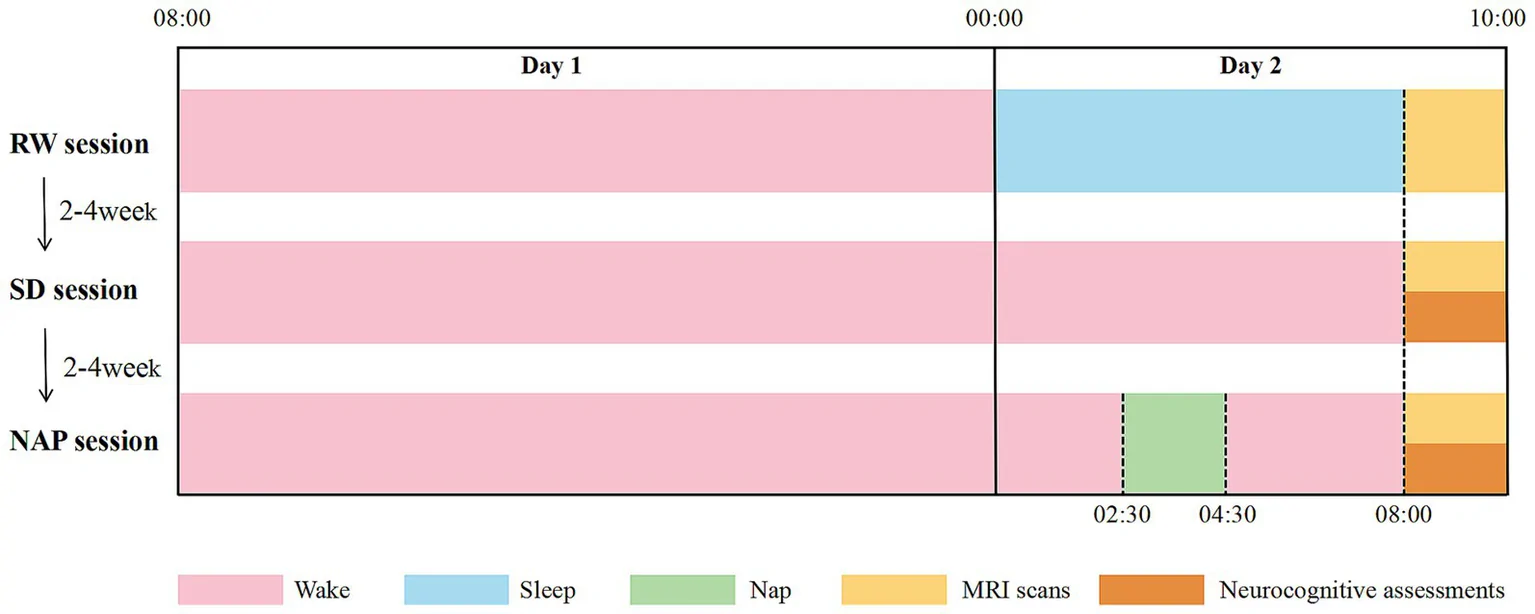
Schematic overview of the experimental design and session procedures.

The study followed a protocol comprising three sessions: Rested Wakefulness (RW), SD, and NAP (Figure 1). Sessions were separated by 2–4 weeks. Participants were not assigned night shifts and were required to maintain a regular sleep schedule for at least 2 weeks prior to the RW session and during all inter-session intervals. The procedures of each session were as follows: For all three sessions, participants remained awake from 08:00 until 00:00 on the preceding day. During the RW session, nurses were allowed a normal night’s sleep from 00:00 to 08:00. In the SD session, participants remained awake in the hospital from 00:00 to 08:00. In the NAP session, participants remained awake from 00:00 to 02:30 and from 04:30 to 08:00, with a scheduled nap (up to 2 h) between 02:30 and 04:30. Actual sleep duration during the nap opportunity was continuously monitored using an electronic wristband. The two-hour duration for napping was based on clinical practicality and existing evidence (20, 21). Neurocognitive assessments and MRI scans were performed between 08:00 and 10:00 in all three sessions. Throughout the SD and NAP sessions, participants were continuously supervised by two researchers during all wakefulness periods and were restricted to low-intensity activities (e.g., reading or patient monitoring), while strenuous physical activity and caffeinated beverages were prohibited. The flowchart of experimental procedure is shown in Figure 1.

### Neurocognitive assessment

Neurocognitive performance was assessed using a battery of standardized tests, including the Digit Symbol Test (DST), Number Connection Test A (NCT-A), Number Connection Test B (NCT-B), Digit Span Test (DS), and the California Verbal Learning Test, Second Edition (CVLT-II). The DST was used to assess processing speed, visuomotor coordination, and sustained attention (22). The NCT-A primarily reflects visual scanning ability and psychomotor speed, and the NCT-B additionally assesses executive function and cognitive flexibility due to task-switching demands (22). The DS evaluates short-term and working memory capacity (23). Verbal learning and episodic memory were assessed using the CVLT-II, which measures immediate learning and delayed recall performance across multiple trials (24). A detailed description of the CVLT-II has been provided elsewhere (11).

### MRI data acquisition

MRI data were acquired using a 3T scanner (Prisma; Siemens Medical Systems, Erlangen, Germany) equipped with a 64-channel head coil. Resting-state functional images were obtained using an echo planar imaging sequence with the following parameters: repetition time = 700 ms, echo time = 30 ms, flip angle = 50°, matrix = 76 × 76, field of view = 228 mm × 228 mm, slice thickness = 3 mm, and 48 contiguous axial slices. Spatial normalization of resting-state functional images was performed using high-resolution three-dimensional T1-weighted images (1 × 1 × 1 mm), acquired with a magnetization-prepared rapid gradient echo sequence. Subjects were instructed to remain still with their eyes closed (but not to fall asleep) and avoid engaging in specific thoughts during the scan.

### MRI data analysis

Functional data preprocessing was conducted using the Data Processing & Analysis for Brain Imaging (DPABI v6.0; http://rfmri.org/dpabi) and Statistical Parametric Mapping software (SPM 12; https://www.fil.ion.ucl.ac.uk/spm/). The preprocessing steps included: discarding the initial 30 volumes (for scanner calibration and adaptation of the participants to the scanning environment); realignment; spatial normalization; smoothing with a full-width at half maximum of 4 mm; linear detrending; temporal band-pass filtering (0.01–0.08 Hz); and regressing out nuisance covariates, such as motion parameters estimated using the Friston-24 model, as well as mean signals from cerebrospinal fluid, white matter, and the entire brain. Participant head motion was assessed using mean frame-wise displacement (FD), calculated according to Jenkinson’s formula (a measure corresponding to the temporal derivative of motion parameters (25)). An eligibility threshold of mean FD < 0.2 mm was applied (26).

VMHC computation was performed using DPABI, following these steps (15): (1) A mean structural image was created by averaging the normalized T1-weighted images of all subjects. (2) A group-specific symmetrical template was generated by averaging the mean structural image with its left–right mirrored version, excluding the brain’s midline area. (3) The normalized T1-weighted images were registered to the symmetric template, and the corresponding non-linear transformation was applied to the normalized functional images. (4) Pearson’s correlation coefficients were calculated between the time series of each voxel and its corresponding mirrored voxel in the contralateral hemisphere. It is important to note that this calculation was masked using a gray matter segmentation of the group-specific symmetric T1 template, thresholded at 20% (15, 16, 27). (5) Fisher’s z-transformation was applied to convert the correlation coefficients into z-values, referred to as VMHC per voxel. The global VMHC value was computed as the mean VMHC across all voxels within the gray matter mask.

### Statistical analysis

The normality of all variables was evaluated using the Shapiro–Wilk test. For variables conforming to a normal distribution (i.e., global VMHC across three sessions), repeated-measures analysis of variance (ANOVA) was performed, followed by *post hoc* paired-samples *t*-tests for pairwise comparisons between the RW and SD, RW and NAP, and SD and NAP sessions. In contrast, for neurocognitive assessment outcomes that deviated from normal distribution, the nonparametric Wilcoxon test was adopted to compare performance discrepancies between the SD and NAP sessions. Given the multiple pairwise comparisons across cognitive outcome metrics, false discovery rate (FDR) correction was applied to control for type I error risk induced by multiple testing. In addition, changes in global VMHC and neurocognitive measurements between the SD and NAP sessions were calculated. Spearman’s correlation analyses were then performed to assess their relationships, with statistical significance set at *p* < 0.05.

Voxel-wise statistical analyses of the VMHC values were conducted using DPABI software. A one-sample *t*-test was used to assess intra-session VMHC patterns. To examine VMHC differences between session pairs, *post hoc* paired comparisons were performed following a significant repeated-measures ANOVA, to compare the VMHC between the RW and SD, RW and NAP, and SD and NAP sessions. Mean FD was included as a covariate. Statistical significance was assessed using the threshold-free cluster enhancement method for multiple comparison corrections, with a family-wise error (FWE)-corrected threshold set at *p* < 0.05. Additionally, despite meeting stringent FWE correction criteria, extremely small clusters were excluded based on a size threshold of 10 voxels.

## Results

All participants successfully completed the nap session. The mean actual sleep duration during the nap opportunity was 111.7 ± 9.3 min (range: 91–120 min). Table 1 presents the results of the neurocognitive assessment. Compared to the SD session, participants in the NAP session showed a significant increase in the number of correct responses on the DST (69.7 ± 8.4 vs. 71.5 ± 7.4, *P_corrected_* = 0.046) and DS (14.3 ± 2.4 vs. 16.2 ± 2.5, *P_corrected_* = 0.012). They also required less time to complete the NCT-A (21.1 ± 4.7 vs. 18.0 ± 5.1, *P_corrected_* = 0.033). Additionally, compared to the SD session, participants in the NAP session showed a significant increase in total correct responses on CVLT¬-II trials I–V (67.0 ± 6.3 vs. 72.0 ± 7.4, *P_corrected =_* 0.012). Significant improvements were also observed in the number of correct responses on the CVLT-¬II short-delay cued recall trial (14.2 ± 2.0 vs. 15.0 ± 1.2, *P_corrected_* = 0.033), long-delay free recall trial (14.4 ± 1.7 vs. 15.2 ± 1.1, *P_corrected_* = 0.022), and long-delay cued recall trial (14.3 ± 2.0 vs. 15.2 ± 1.0, *P_corrected_* = 0.027).

**Table 1:** Neurocognitive assessment results.

Neurocognitive assessments	SD session	NAP session	*P_FDR-corrected_*
DST (correct number)	69.7 ± 8.4	71.5 ± 7.4	**0.046**
NCT-A (time, s)	21.1 ± 4.7	18.0 ± 5.1	**0.033**
NCT-B (time, s)	33.5 ± 6.9	31.2 ± 7.5	0.270
DS (correct number)	14.3 ± 2.4	16.2 ± 2.5	**0.012**
CVLT-II			
Trials I–V (total correct number)	67.0 ± 6.3	72.0 ± 7.4	**0.012**
Short-delay free recall (correct number)	14.4 ± 2.0	14.8 ± 1.6	0.101
Short-delay cued recall (correct number)	14.2 ± 2.0	15.0 ± 1.2	**0.033**
Long-delay free recall (correct number)	14.4 ± 1.7	15.2 ± 1.1	**0.022**
Long-delay cued recall (correct number)	14.3 ± 2.0	15.2 ± 1.0	**0.027**

DST, digit symbol test; NCT-A, Number Connection Tests A; NCT-B, Number Connection Tests B; DS, Digit Span Test; CVLT-II, California Verbal Learning Test, Second Edition. Bold text indicates *p* < 0.05.

**Figure 2:**
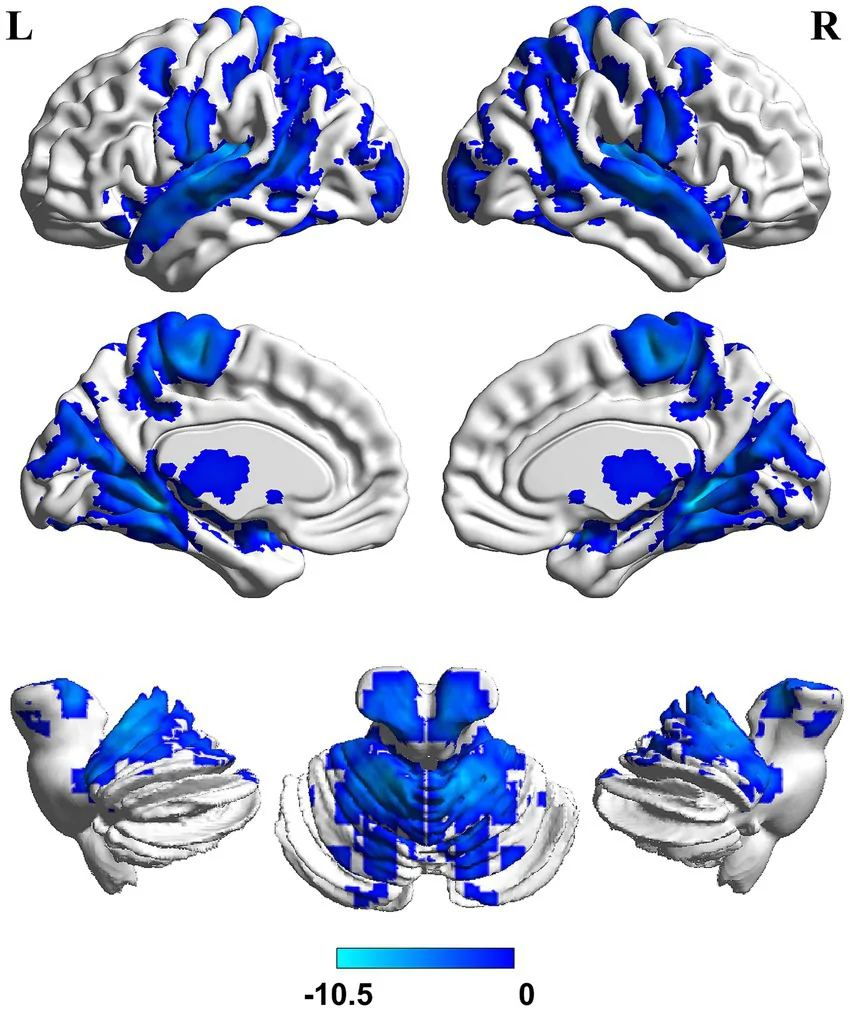
Brain regions with significantly increased VMHC in SD session compared to that in the RW session. Color bar represents the *T* value. L, left; R, right; RW, rested wakefulness; SD, sleep deprivation.

**Figure 3:**
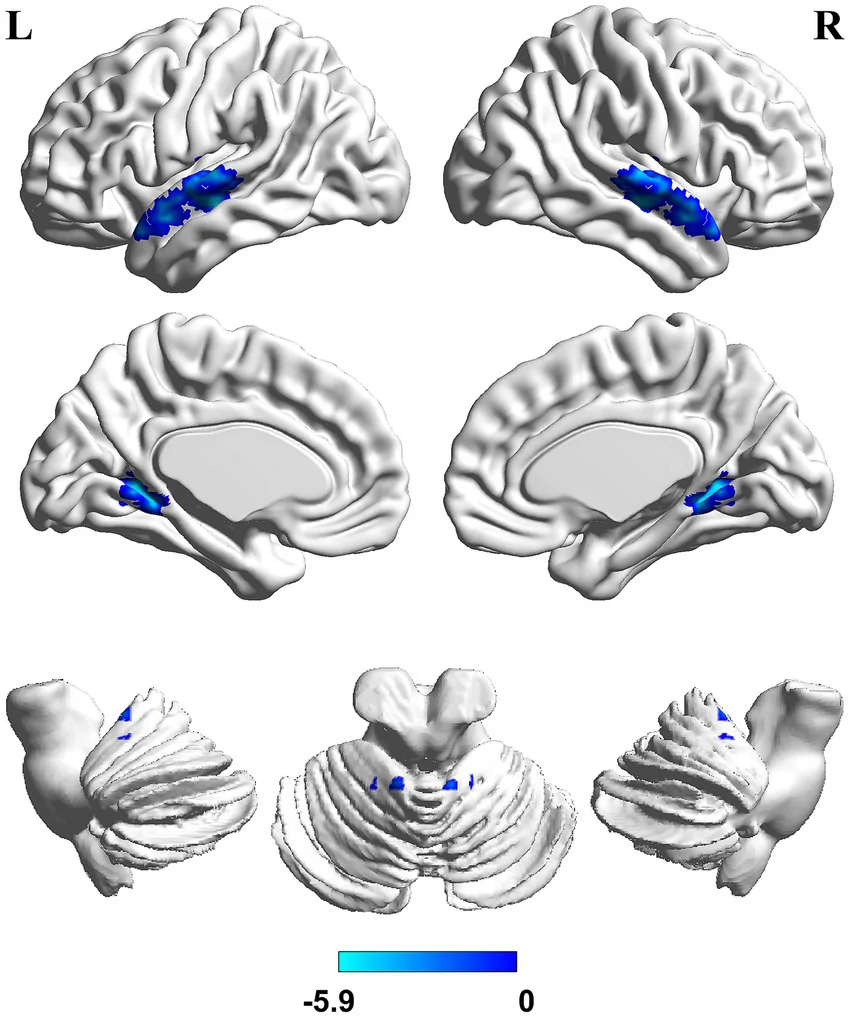
Brain regions with significantly increased VMHC in NAP session compared to that in the RW session. Color bar represents the *T* value. L, left; R, right; RW, rested wakefulness; NAP, napping.

**Figure 4:**
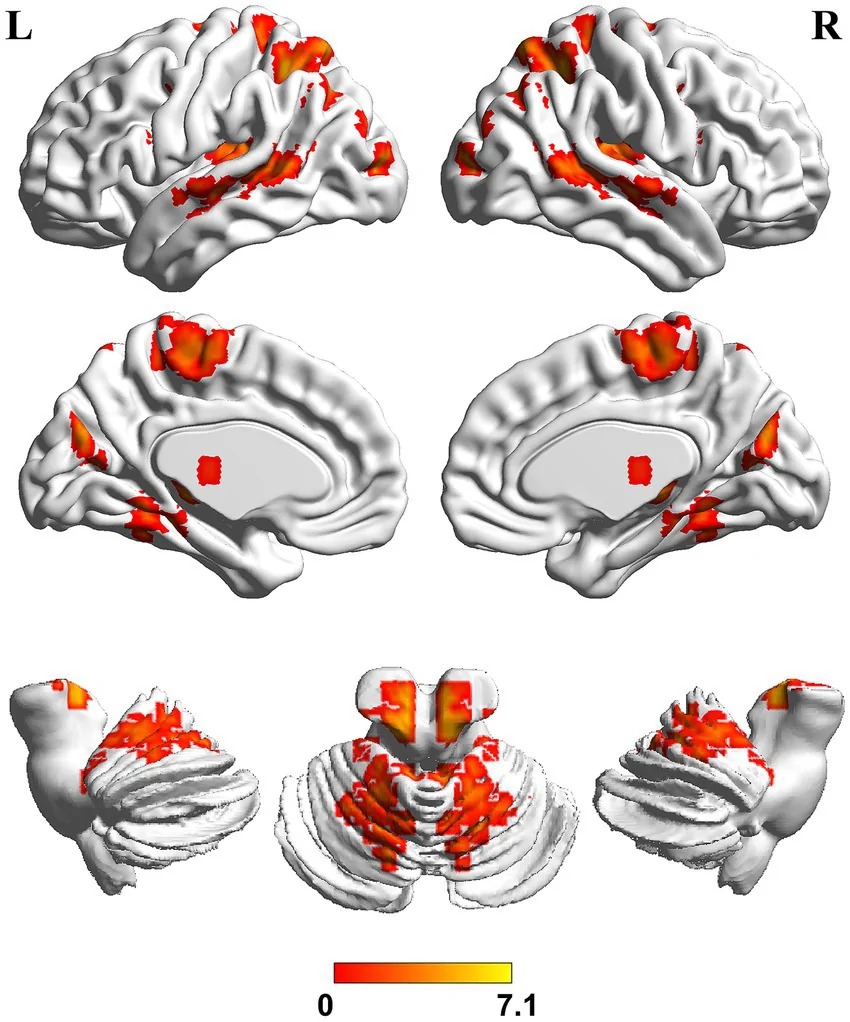
Brain regions with significantly decreased VMHC in NAP session compared to that in the SD session. Color bar represents the *T* value. L, left; R, right; RW, rested wakefulness; SD, sleep deprivation; NAP, napping.

The profiles of global VMHC for each session are shown in Supplementary Figure 1. Brain regions with significant VMHC differences across the three sessions are displayed in Supplementary Figure 2. Specifically, compared to RW, SD resulted in increased VMHC values in extensive brain regions (Figure 2; Table 2), primarily involving the bilateral superior, middle, and inferior temporal gyri; temporal pole; Heschl’s gyrus; hippocampus; parahippocampal gyrus; amygdala; fusiform gyrus; superior, middle, and inferior occipital gyri; lingual gyrus; calcarine; cuneus; precuneus; posterior cingulate gyrus; angular gyrus; supramarginal gyrus; superior and inferior parietal gyri; postcentral gyrus; paracentral lobule; precentral gyrus; supplementary motor area; middle and inferior frontal gyri; Rolandic operculum; insula; thalamus; caudate nucleus; putamen; pallidum; and superior parts of the cerebellum. Compared to RW, NAP led to increased VMHC values in the bilateral superior and middle temporal gyri, temporal pole, lingual gyrus, calcarine, and superior parts of the cerebellum (Figure 3; Table 3). Compared to SD, NAP resulted in decreased VMHC values in several brain regions, primarily including the bilateral superior and middle temporal gyri, Heschl’s gyrus, parahippocampal gyrus, fusiform gyrus, middle occipital gyrus, lingual gyrus, cuneus, angular gyrus, superior and inferior parietal gyri, postcentral gyrus, paracentral lobule, precentral gyrus, supplementary motor area, inferior frontal gyrus, Rolandic operculum, insula, thalamus, putamen, and superior parts of the cerebellum (Figure 4; Table 4).

**Table 2:** Regions showing significantly increased VMHC in the SD session compared to that in the RW session.

Cluster index	Cluster size (voxels)	Regions	Brodmann area	Peak MNI coordinates			Peak *T* value
				*x*	*y*	*z*	
1	6,550	Calcarine fissure and surrounding cortex	17	24	−57	15	−10.46
		Superior temporal gyrus	22				
		Middle temporal gyrus	21				
		Inferior temporal gyrus	20				
		Temporal pole: superior temporal gyrus	38				
		Temporal pole: middle temporal gyrus	38				
		Heschl gyrus	41				
		Hippocampus	36				
		Parahippocampal gyrus	36				
		Fusiform gyrus	37				
		Superior occipital gyrus	19				
		Middle occipital gyrus	18				
		Inferior occipital gyrus	17				
		Lingual gyrus	37				
		Cuneus	18				
		Precuneus	7				
		Posterior cingulate gyrus	31				
		Angular gyrus	39				
		Supramarginal gyrus	40				
		Superior parietal gyrus	7				
		Inferior parietal gyrus	40				
		Postcentral gyrus	3				
		Paracentral lobule	4				
		Precentral gyrus	4				
		Middle frontal gyrus	10				
		Inferior frontal gyrus, opercular part	44				
		Inferior frontal gyrus, orbital part	47				
		Supplementary motor area	6				
		Rolandic operculum	44				
		Insula	13				
		Amygdala					
		Thalamus					
		Caudate nucleus					
		Putamen					
		Pallidum					
		Cerebelum_3					
		Cerebelum_4_5					
		Cerebelum_6					
		Vermis_4_5					
		Vermis_6					
		Cerebelum_Crus1					

VMHC, voxel-mirrored homotopic connectivity; RW, rested wakefulness; SD, sleep deprivation; MNI, Montreal Neurological Institute.

All identified VMHC clusters are annotated according to the right hemisphere parcellation scheme of the Automated Anatomical Labeling (AAL) template and associated Brodmann areas.

**Table 3:** Regions showing significantly increased VMHC in the NAP session compared to that in the RW session.

Cluster index	Cluster size (voxels)	Regions	Brodmann area	Peak MNI coordinates			Peak *T* value
				*x*	*y*	*z*	
1	153	Superior temporal gyrus	22	51	−18	−6	−5.98
		Middle temporal gyrus	21				
		Temporal pole: superior temporal gyrus	38				
		Temporal pole: middle temporal gyrus	38				
2	63	Lingual gyrus	37	21	−45	−6	−5.39
		Calcarine fissure and surrounding cortex	17				
		Cerebelum_4_5					

VMHC, voxel-mirrored homotopic connectivity; RW, rested wakefulness; MNI, Montreal Neurological Institute.

All identified VMHC clusters are annotated according to the right hemisphere parcellation scheme of the Automated Anatomical Labeling (AAL) template and associated Brodmann areas.

**Table 4:** Regions showing significantly decreased VMHC in the NAP session compared to that in the SD session.

Cluster index	Cluster size (voxels)	Regions	Brodmann area	Peak MNI coordinates			Peak *T* value
				*x*	*y*	*z*	
1	393	Superior temporal gyrus	22	33	−30	15	5.41
		Middle temporal gyrus	21				
		Heschl gyrus	41				
		Rolandic operculum	44				
		Insula	13				
		Putamen					
2	24	Middle temporal gyrus	22	48	−57	15	3.67
3	228	Parahippocampal gyrus	36	6	−45	−12	5.58
		Fusiform gyrus	37				
		Lingual gyrus	37				
		Cerebelum_4_5					
		Cerebelum_6					
		Vermis_4_5					
4	17	Middle occipital gyrus	18	30	−93	12	5.49
5	29	Middle occipital gyrus	18	36	−84	27	5.49
6	28	Cuneus	18	3	−75	24	4.64
7	233	Inferior parietal gyrus	40	36	−51	54	5.96
		Angular gyrus	39				
		Superior parietal gyrus	7				
8	181	Supplementary motor area	6	6	−15	63	4.76
		Postcentral gyrus	3				
		Paracentral lobule	4				
		Precentral gyrus	4				
9	49	Precentral gyrus	4	51	6	36	4.79
		Inferior frontal gyrus, opercular part	44				
10	104	Thalamus		6	−18	12	7.08

VMHC, voxel-mirrored homotopic connectivity; SD, sleep deprivation; MNI, Montreal Neurological Institute.

All identified VMHC clusters are annotated according to the right hemisphere parcellation scheme of the Automated Anatomical Labeling (AAL) template and associated Brodmann areas.

**Figure 5:**
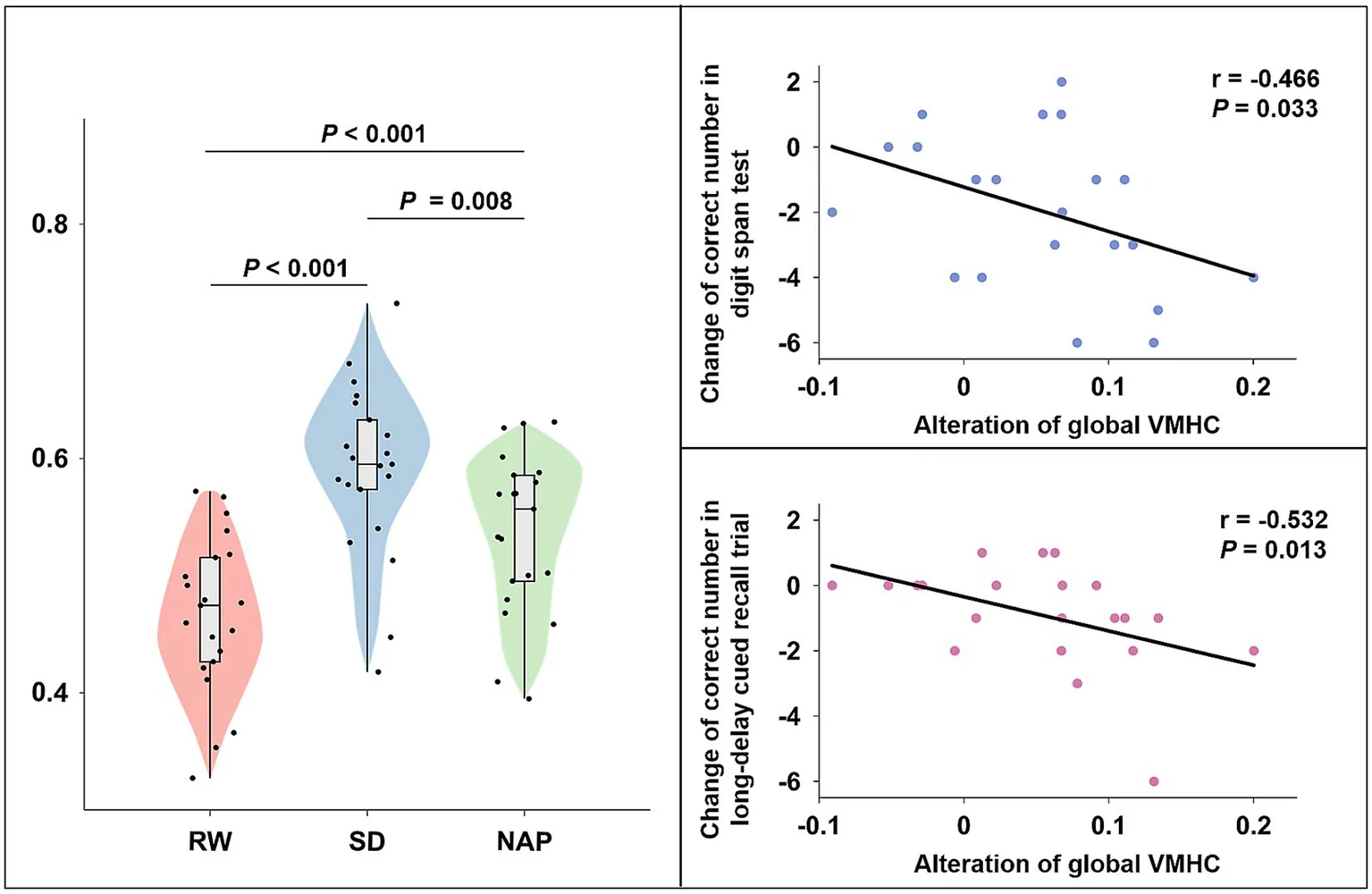
Global VMHC differences among sessions, and correlations between alterations in global VMHC and changes in neurocognitive performance between SD and NAP sessions. RW, rested wakefulness; SD, sleep deprivation; NAP, napping.

Figure 5 illustrates the significant differences in global VMHC values across the three sessions (RW: 0.466 ± 0.068; SD: 0.590 ± 0.073; NAP: 0.537 ± 0.069). Compared to RW, both SD and NAP showed significantly increased global VMHC (all *p* < 0.001), whereas NAP exhibited a significant decrease in global VMHC compared to SD (*p* = 0.008). Between the SD and NAP sessions, the change in global VMHC was negatively correlated with the change in the number of correct responses on the DS (*r* = −0.466, *p* = 0.033). Similarly, the alteration in global VMHC was negatively correlated with the change in the number of correct responses on the CVLT-II long-delay cued recall trial (*r* = −0.532, *p* = 0.013).

## Discussion

The present study investigated alterations in whole-brain homotopic functional coordination in night shift nurses experiencing SD and NAP conditions. The main findings are summarized as follows: (1) Neurocognitive performance was significantly improved during NAP compared to SD, indicating enhancements in cognitive functions such as visual search, perceptual efficiency, graphomotor and psychomotor speed, executive function, verbal learning, and memory. (2) SD induced substantial alterations in interhemispheric functional coordination, as evidenced by a significant increase in VMHC, primarily in bilateral regions of the temporal, occipital, and parietal cortices, sensorimotor areas, middle and inferior frontal cortices, insula, subcortical structures, and cerebellum. (3) NAP resulted in increased VMHC in a limited set of regions, including portions of the bilateral temporal cortex, occipital cortex, and cerebellum. (4) Compared with the SD condition, NAP caused a decrease in VMHC across a broad range of brain regions that had previously exhibited elevated VMHC associated with SD, further supporting the restorative impact of NAP on interhemispheric coordination. (5) Our exploratory analyses revealed associations between NAP-related changes in global VMHC and improvements in neurocognitive performance.

SD led to a significant increase in VMHC across extensive brain regions involved in vision, somatosensory processing, motor functions, auditory perception, and higher-order cognition. Consistent with previous studies, the elevated VMHC following SD has been observed in brain areas related to visual processing, sensorimotor integration, auditory function, default-mode operations, and subcortical networks (18, 28). The enhancement of interhemispheric functional connectivity may act as a compensatory mechanism to counteract the adverse effects of SD. Such adaptation may be crucial for maintaining brain function during periods of reduced arousal. Supporting this interpretation, a prior study found an increased diversity of connector hubs in brain networks, suggesting a compensatory response to SD (29). Furthermore, this idea is reinforced by transcriptomic research showing upregulation of genes related to energy metabolism and synaptic strengthening during short-term SD (30).

Our findings suggest that NAP exerts a substantial restorative effect on interhemispheric functional coordination, as reflected by a significant reduction in VMHC across regions involved in both higher-order cognition and primary sensory/motor processing. Compared with SD, NAP reduced VMHC in regions associated with working memory and verbal episodic memory, including the opercular part of the inferior frontal gyrus, angular gyrus, superior parietal gyrus, and parahippocampal gyrus (31, 32). These VMHC changes were accompanied by improvements in corresponding cognitive performance, as evidenced by increased scores on DS and the CVLT-II long-delay cued recall trial (11). Consistent with our findings, previous fMRI research has shown that daytime napping following SD partially alleviates disrupted functional connectivity and cognitive function (33, 34), potentially through sleep-dependent memory consolidation and protein synthesis (35). NAP also reduced VMHC in regions subserving vision, somatosensory processing, motor function, and auditory perception, suggesting improved interhemispheric integration of primary sensory and motor information that may facilitate the recovery of higher-order cognitive functions (36). Supporting this view from a molecular perspective, evidence from a study on visual discrimination task training demonstrates that sleep-dependent memory consolidation is also associated with alterations in the protein synthesis rate of the primary visual cortex (35). The restoration effect of NAP on interhemispheric coordination may also involve neuroendocrine mechanisms. By partially alleviating circadian disruption induced by SD, NAP may promote the recovery of melatonin secretion, which has been associated with interhemispheric functional connectivity (28, 37). Thus, the restoration of melatonin balance likely contributes to the beneficial effects of NAP on interhemispheric functional coordination.

Nevertheless, despite marked VMHC reductions in most SD-affected regions after NAP, several regions (such as bilateral temporal and occipital cortices) remained significantly elevated relative to RW. This residual elevation indicates that a 2-h nap provided substantial but incomplete recovery. The persistence may reflect either a residual carry-over effect of the preceding sleep loss or an adaptive functional configuration that temporarily sustains essential sensory and cognitive processing during recovery, consistent with previous evidence that enhanced interhemispheric connectivity following SD serves a compensatory role (18). Hence, although NAP substantially alleviated SD-related alterations, complete normalization of interhemispheric coordination likely necessitates a longer restorative sleep period.

Our findings must be interpreted in light of several limitations. First, the exploratory design and modest sample of young female nurses constrain the generalizability of our results to older or more experienced nurses and to male populations, who may exhibit different physiological and cognitive responses to SD and NAP. Second, to reduce potential order effects, practice effects, and carry-over effects on cognitive measures, future studies should implement fully counterbalanced designs and control assessments to mitigate these confounders. Third, while we used VMHC to assess interhemispheric functional coordination by aligning functional images with a group-specific structural symmetrical template, it is important to acknowledge that human brains are not perfectly symmetrical. Therefore, potential morphometric asymmetry may influence the interpretation of VMHC measurements. Fourth, brain fMRI was conducted only on the morning following NAP, limiting our ability to assess the long-term effects of NAP on interhemispheric functional coordination. Future longitudinal studies are needed to investigate the duration of NAP-related functional restoration and its relationship with neurocognitive performance. Fifth, sleep–wake status during MRI scanning were assessed solely by self-report. Future studies should incorporate objective monitoring (e.g., EEG) to more reliably verify wakefulness.

Our findings demonstrate that SD disrupts interhemispheric functional coordination, across brain regions involved in vision, somatosensory and motor processing, auditory perception, and cognitive functions in nurses. Night-shift napping appears to reorganize interhemispheric functional connections. Our preliminary data link this neural reorganization to cognitive improvement in sleep-deprived nurses, although these findings are limited in scope and require validation in larger cohorts.

## Data Availability

The raw data supporting the conclusions of this article will be made available by the authors, without undue reservation.

## Supplementary material

SUPPLEMENTARY FIGURE 1 VMHC pattern in RW, SD, and NAP sessions. Color bar represents the T value. L, left; R, right; RW, rested wakefulness; SD, sleep deprivation; NAP, napping.

SUPPLEMENTARY FIGURE 2 Brain regions showing significant differences in VMHC value among the three sessions. Color bar represents the F value. L, left; R, right.
